# Mortality in an Aboriginal Medical Service (Redfern) cohort

**DOI:** 10.1186/1478-7954-11-2

**Published:** 2013-02-07

**Authors:** Stephen Morrell, Bronwen Phillips, Richard Taylor, John Daniels, Kate Burgess, Naomi Mayers

**Affiliations:** 1 School of Public Health and Community Medicine, University of NSW, Kensington, NSW, Australia; 2 Aboriginal Medical Service, Redfern, Australia

## Abstract

**Background:**

Published estimates of Aboriginal mortality and life expectancy (LE) for the eastern Australian states are derived from demographic modelling techniques to estimate the population and extent of under-recording of Aboriginality in death registration. No reliable empirical information on Aboriginal mortality and LE exists for New South Wales (NSW), the most populous Australian state in which 29% of Aboriginal people reside.

This paper estimates mortality and LE in a large, mainly metropolitan cohort of Aboriginal clients from the Aboriginal Medical Service (AMS) Redfern, Sydney, NSW.

**Methods:**

Identifying information from patient records accrued by the AMS Redfern since 1980 of definitely Aboriginal clients, without distinction between Aboriginal and Torres Strait Islander (n=24,035), was extracted and linked to the National Death Index (NDI) at the Australian Institute of Health and Welfare (AIHW). Age-specific mortality rates and LEs for each sex were estimated using the AMS patient population as the denominator, discounted for deaths. Directly age-standardised mortality and LEs were estimated for 1995–1999, 2000–2004 and 2005–2009, along with 95% confidence intervals. Comparisons were made with other estimates of Aboriginal mortality and LE and with the total Australian population.

**Results:**

Mortality declined in the AMS Redfern cohort over 1995–2009, and the decline occurred mostly in the ≤44 year age range. Male LE at birth was estimated to be 64.4 years (95%CI:62.6-66.1) in 1995–1999, 65.6 years (95%CI:64.1-67.1) in 2000–2004, and 67.6 years (95%CI:65.9-69.2) for 2005–2009. In females, these LE estimates were 69.6 (95%CI:68.0-71.2), 71.1 (95%CI:69.9-72.4), and 71.4 (95%CI:70.0-72.8) years. LE in the AMS cohort was 11 years lower for males and 12 years lower for females than corresponding all-Australia LEs for the same periods. These were similar to estimates for Australian Aboriginal people overall for the same period by the Aboriginal Burden of Disease for 2009, using the General Growth Balance (GGB) model approach, and by the Australian Bureau of Statistics (ABS) for 2005–2007. LE in the AMS cohort was somewhat lower than these estimates for NSW Aboriginal people, and higher than ABS 2005–2007 estimates for Aboriginal people from Northern Territory, South Australia, and Western Australia.

**Conclusions:**

The AMS Redfern cohort has provided the first empirically based estimates of mortality and LE trends in a large sample of Aboriginal people from NSW.

## Introduction

Until the late 1990s, mortality in Australian Aboriginal people was incompletely recorded, especially outside Western Australia (WA), South Australia (SA), and the Northern Territory (NT). Improvements in some aspects of mortality in NT Aboriginals have been documented, especially in child and infant mortality from the 1960s [1] and 1970s [2-5].

In jurisdictions outside WA, SA, and NT, under-recording of Aboriginal status in both death registration and the population census has made it difficult to ascertain the extent of Aboriginal mortality differences in other parts of the country; and the use of differing estimation methods over time has made determining secular trends virtually impossible. The Australian Bureau of Statistics (ABS) has used a number of indirect and direct methods for estimating Aboriginal mortality and LE. However, no empirically based estimates of Aboriginal mortality or LE exist for the eastern Australian states, where nearly two-thirds of Aboriginal people live, or for New South Wales (NSW) where 29% of Australian Aboriginal people live [6]. A consequence of this uncertainty is a tendency to ascribe mortality and LE deficits in Aboriginal people compared to other Australians either from differences from earlier ABS mortality estimates or from estimates of Aboriginal mortality for the NT [7].

In this paper, we present estimates of Aboriginal mortality based on a large cohort of Aboriginal Medical Service (AMS) Redfern patients (n=24,035) who first attended the service since between 1980 and 2009. The cohort is exclusively Aboriginal and/or Torres Strait Islander and overwhelmingly comprises NSW residents, many of whom live in metropolitan areas. We estimate mortality and LE and their secular trends in this cohort and compare these with estimates for other Aboriginal/Torres Strait Islander populations and to the Australian population as a whole.

## Methods

### Sample

The AMS cohort for this study comprised clients definitely identified as Aboriginal or Torres Strait Islander who first presented to the AMS from 1980 to 2009 (n=24,035). This represents 16% of the estimated Aboriginal and Torres Strait Islander population of NSW (n=148,178, as derived from the 2006 census) [8].

Some AMS clients are Pacific Island or Maori people, and were excluded from the study. The AMS Redfern cohort was largely metropolitan: 75% were last recorded as living in a metropolitan Sydney postcode, with an additional 2.4% last recorded as living in a postcode from the Central Coast, Wollongong, or Newcastle. 17% were last recorded as living in NSW outside the Sydney-Newcastle-Wollongong conurbation, 3.9% as living outside NSW, and 1.7% with missing postcode. The cohort was thus overwhelmingly from NSW (> 94%). However, the medical records did not reliably differentiate Torres Strait Islander versus Aboriginal (or both) status.

### Data and linkage

Identifying information including names, dates of birth, addresses, and dates of last AMS presentation were extracted from paper and electronic patient records of the AMS Redfern. These were linked to the Australian National Death Index (NDI) operated by the Australian Institute of Health and Welfare (AIHW). Death data held on the NDI include all deaths registered in Australia from 1980 onwards; this precluded using the cohort from the inception of the AMS in 1971 for matching AMS clients definitely known to be Aboriginal (n=32,058). A consequence was the rendering of mortality and LE estimates for 1980–1994 unreliable due to initial small numbers. Of the 24,035 AMS clients who first presented to the AMS in 1980 or after, as used in the present study, 14,969 (62%) first presented to the AMS in 1990 or later.

The algorithm for linking of AMS clients to the NDI was probabilistic and developed in-house by the AIHW [9]. Differing weights were assigned to linking fields to yield overall scores reflecting the probability of a match. Several passes of the AMS data over NDI mortality records were made with altered weighting factors and linking field restrictions. Postcode information was also used in further passes to increase the chances of valid matches. Following each data pass in the linkage process, an extensive clerical review was conducted to resolve probable and possible matches into definite matches or non-matches. The remaining possible/probable records were then re-linked using changed restrictions and weightings.

The first pass of data linkage required matching on exact date of birth and surname, and for date of death occurring after date of last known AMS appearance; allowance was made for some variation on other fields such as first name(s). Certain matches were ascertained on the basis of an exact match of date of birth and surname and a high matching score on the remaining fields. Following clerical review, further certain matches were found, along with certain non-matches. These were deleted from the file of probable/possible matches, and the remainder was then re-linked against relaxed date-of-birth criteria with remaining matching field conditions unaltered. This process was repeated against alterations in matching criteria eight times to minimise the number of probable/possible matches and thereby maximise the number of certain matches.

Some AMS clients were known to the AMS to be deceased but were not linkable to the NDI (n=102). These comprised 10% of all mortality used in the analyses, and illustrate the difficulties of matching records with multiple names (forenames and surnames) and multiple dates of birth. In order for the matching to be performed by the NDI, individual records in the AMS cohort had to be repeated several times to reflect the multiplicity of identifying information on individuals, despite the NDI algorithm being able to accommodate several aliases. Some AMS clients had more than four aliases, none of which matched NDI mortality records, and some had three dates of birth. Furthermore, a number of the clients known to be deceased by the AMS most probably were registered under an identity not known to the AMS.

From a total of 22,768 possible matches returned by the NDI, 3,033 were certain or probable and 772 considered possible but not accepted. In total, 616 male and 408 female deaths in the cohort were ascertained (Table 1).

**Table 1:** Estimated aboriginal mortality and life expectancy at birth, males and females, Aboriginal Medical Service Redfern, 1995-2009

**Period**	**Males**			**Females**		
**MORTALITY**						
	**Deaths**	**Mortality per 1,000**^†^	**95% CI**	**Deaths**	**Mortality per 1,000**^†^	**95% CI**
1995-99	190	10.5	9.0-12.3	109	9.1	7.4-10.9
2000-04	208	11.9	10.4-13.6	137	8.3	7.1-9.7
2005-09	218	8.6	7.6-9.8	162	7.7	6.7-8.8
**LIFE EXPECTANCY AT BIRTH**						
	**Person years**	**Life Expectancy (yrs)**	**95% CI**	**Person years**	**Life Expectancy (yrs)**	**95% CI**
1995-99	27,188	64.4	62.6-66.1	32,436	69.6	68.0-71.2
2000-04	33,382	65.6	64.1-67.1	39,866	71.1	69.9-72.4
2005-09	38,849	67.6	65.9-69.2	46,082	71.4	70.0-72.8

^†^Rate per 1,000 person years, directly age-standardised to the Australian Estimated Resident Population for 30 June 2001 population, based on the 2001 Census of Population and Housing^[10]^.

Denominators for estimating mortality rates and LE study were calculated by cumulating AMS patients for each year, ageing them accordingly, and subtracting any deaths that occurred in the previous year. Starting in 1980, all patients of a given age who first presented to the AMS in 1980 were the denominator for that age. For the 1981 denominator, these 1980 patients were aged by one year, and any who died during 1980 were subtracted from their number; all new patients presenting to the AMS for the first time in 1981 were then added to their number to produce the denominator for 1981. And so on.

### Statistical analysis

Age-specific mortality rates were calculated by 10-year age group to age 65–74 years. Directly age-standardised mortality rates were calculated based on the 2001 Australian Estimated Resident Population as standard [10]. Direct age standardisation and life table analysis minimise artefactual differences in mortality stemming from differences or changes in age structure of the population under study. Life tables [11] were based on five-year age groups to ≥75 years. 95% confidence intervals for age-specific mortality rates in the cohort were based on the Poisson distribution. 95% confidence intervals for directly age-standardised mortality rates [12] were based on the weighted Poisson method of Dobson *et al*[13]. Life tables were calculated to estimate LE at birth in the AMS cohort, along with 95% confidence intervals calculated using the method of Chiang [11]. Trends in sex-specific mortality were tested by negative binomial regression of age-specific counts of deaths (offset by the log of the denominator population) against each time interval as a continuous variable after controlling for five-year age group. PROC GENMOD in SAS v.9.2 was used for this purpose. Time trends in estimated LE were compared between the AMS cohort and the Australian population overall by a repeated measures analysis using PROC GLM in SAS v.9.2.

Ethics approvals for this project were granted by the NSW Aboriginal Health and Medical Research Council, the University of Sydney Human Ethics Committee, the NSW Health Ethics Committee, and the AIHW Ethics Committee.

## Results

**Figure 1:**
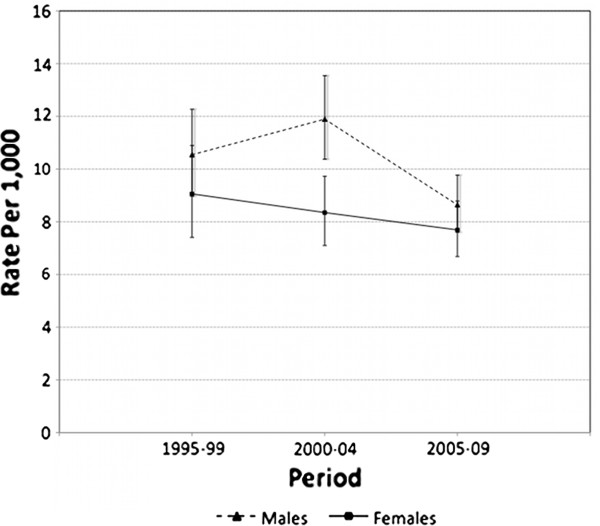
Directly age-standardised mortality, AMS Redfern cohort, males and females, 1995–2009.

The directly age-standardised mortality rate in AMS Redfern cohort males was estimated to be 10.5 (95%CI:9.0-12.3) per 1,000 for 1995–1999, 11.9 (95%CI:10.4-13.6) for 2000–2004, and 8.6 (95%CI:7.6-9.8) for 2005–2009 (Table 1, Figure 1). Corresponding female mortality was estimated as 9.1 (95%CI:7.4-10.9), 8.3 (95%CI:7.1-9.7), and 7.7 (95%CI:6.7-8.8) per 1,000. Mortality declined more uniformly in females over 1995–2009 than in males. However, from negative binomial regression models of mortality (detail not shown), the declining mortality trend in females (RR=0.90 per five-year period) was close to statistical significance (p=0.074), but in males the adjusted RR estimate of 0.85 per five-year period was significant (p=0.005).

**Figure 2:**
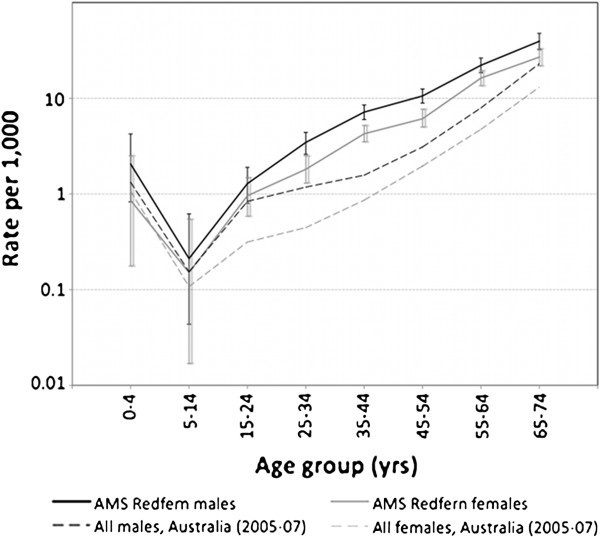
Age-specific mortality, AMS Redfern cohort, 1995–2009, and all Australians (2005–2007), males and females.

**Figure 3:**
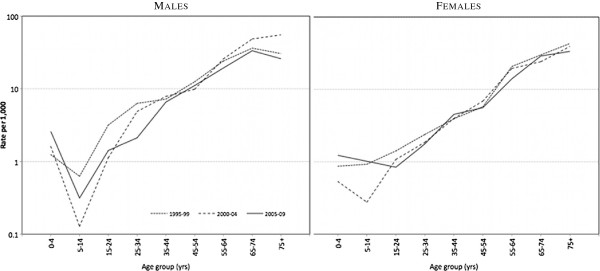
Age-specific mortality, AMS Redfern cohort, 1995–1999, 2000–2004, and 2005–2009, males and females.

By age, AMS cohort mortality is significantly higher than the Australian population from age 25 to 64 years (Figure 2). Mortality declines in the AMS cohort have occurred mainly in the younger 0–44 year age range, more so in males than females (Figure 3). Age-specific mortality in the cohort for 2000–2009 shows that male mortality was significantly higher than females in the 25–54 year age range, as indicated by non-overlapping 95% confidence intervals (Table 2).

**Table 2:** Estimated aboriginal age-specific mortality, males and females, Aboriginal Medical Service Redfern, 2000-2009

**Age group (yrs)**	**Males**			**Females**		
	**Deaths**	**Mortality**^**†**^	**95% CI**	**Deaths**	**Mortality**^**†**^	**95% CI**
0-4	7	2.06	0.83-4.24	3	0.86	0.18-2.51
5-14	3	0.21	0.04-0.62	2	0.15	0.02-0.54
15-24	24	1.28	0.80-1.90	20	0.96	0.59-1.48
25-34	61	3.46	2.60-4.40	41	1.81	1.30-2.50
35-44	141	7.17	6.00-8.50	104	4.26	3.50-5.20
45-54	137	10.58	8.90-12.50	84	6.11	5.00-7.70
55-64	125	22.13	18.50-26.30	118	16.26	13.50-19.40
65-74	101	39.42	32.20-47.70	93	26.94	21.80-32.90
75+	47	37.87	28.00-50.00	68	35.85	27.90-45.20

^†^Rate per 1,000 person years.

**Figure 4:**
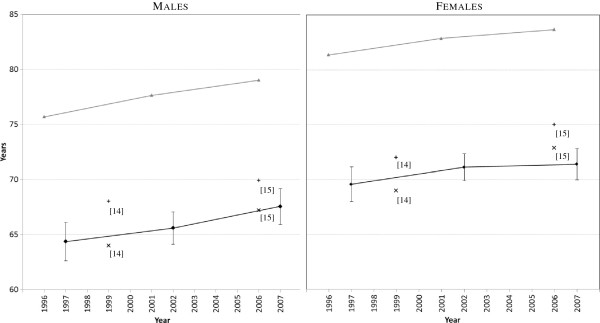
Aboriginal life expectancy at birth (years): AMS Redfern cohort, 1995–1999, 2000–2004, and 2005–2009, compared with Australia and NSW overall.

Male LE was estimated to be 64.4 years (95%CI:62.6-66.1) in 1995–1999, 65.6 years (95%CI:64.1-67.1) in 2000–2004, and 67.6 years (95%CI:65.9-69.2) in 2005–2009 (Table 1 & Figure 4). In females, these estimates were 69.6 (95%CI:68.0-71.2), 71.1 (95%CI:69.9-72.4), and 71.4 (95%CI:70.0-72.8) years. AMS cohort LE estimates were 11.2-11.4 years lower than all-Australia male life expectancies, and 11.8-12.3 years lower for females for the same periods. The AMS cohort LEs were similar to the GGB Aboriginal estimates for Australia (1999) from the report by Vos et al [14], and the ABS 2005–2007 Aboriginal estimate for Australia [15], but somewhat lower than corresponding NSW estimates from these sources.

From the repeated measures analyses (detail not shown), the increasing time trends of LE in both males and females from the AMS cohort were not statistically significant; however, they also were not significantly different from the corresponding LE trends in the Australian male and female populations overall.

## Discussion and conclusions

The present empirical study of mortality and LE in the large sample of mostly metropolitan Aboriginal people comprising the AMS Redfern cohort is similar to indirect estimates for Australian Aboriginal people nationally from the Aboriginal Burden of Disease study (based on 1999 mortality), and the Australian Bureau of Statistics estimates (from 2005–2007 mortality). The LE deficit in the AMS cohort, compared to the Australian population overall, is 11–12 years for 1995–2009. These differences are similar to those of the AMS cohort compared to NSW LE, which in 2006 was three to six months higher than the difference compared to the Australian population overall [16].

From 1967–1971 to 1984–1988, estimates of Aboriginal LE in the jurisdiction with the most complete recording of Aboriginal status at the time, the Northern Territory, showed a four-year increase for males, almost entirely due to a decline in infant (0–1 year) mortality, and an 8.6-year increase in female LE, 50% of which was accounted for by the infant mortality reduction [17]. From 1984–1988 to 2000–2004, NT Aboriginal LE increases (a further 4.0 years in males and 5.5 years in females) came mainly from mortality improvements in adult ages, at 45–64 years in males and ≥55 years in females. From the late 1960s, NT Aboriginal LE increased from 52 years in males and 54 years in females, to 60 and 68 years respectively by 2000–2004 [17]. Despite these well-documented improvements, Aboriginal LE at birth in the NT for 2005–2007, where Aboriginal death registration and denominator populations are well recorded, was estimated by the ABS to be 17.2 and 13.4 years below that of Australian male and female populations respectively [15].

A Western Australian study found that male Aboriginal mortality declined by 3% between 1985–1989 and 1990–1994 compared to 11% in non-Aboriginal males. However, in Aboriginal females mortality actually increased by 11% compared to a 5% decline in non-Aboriginal females [18]. Some increases in Aboriginal mortality in Northern Australia have been attributed to movements of Aboriginal people to outlying settlements [19], although Aboriginal mortality in such areas has been shown to vary *inter alia* according to the adequacy of local health care facilities [20].

A study of Aboriginal settlements in Queensland in 1980 found Aboriginal mortality to vary significantly according to social environment variables, including how the settlements were run by Queensland government administrators [21]. Aboriginal LE in selected rural areas of NSW for 1980 and 1981, based on deaths reported by Aboriginal health workers, was estimated to be 48 years for males and 57 years for females [22].

The time trend of mortality and LE estimates for AMS Redfern differ from these NT and WA trends in significant respects. For instance, most of the mortality improvement in the AMS Redfern cohort has occurred in the male younger age groups to age 35 years, but in females the improvement has not been as consistent and has been confined to younger 0–9 year ages.

Indirect means have been used to estimate Aboriginal mortality and LE for jurisdictions outside WA, SA, and NT where Aboriginal status has been incompletely recorded. From 1991 to 1996, the ABS used a method based on that developed by Preston and Hill to estimate mortality from incomplete death recording [23], which produced estimates of Aboriginal LE for 1991–1996 of 57 years for males and 62 years for females, approximately 20 years below the overall Australian population at the time [24]. However, the Preston-Hill approach assumes stable populations with no net migration, and more importantly does not account for changes in the propensity of Aboriginal people to identify as Aboriginal at successive population censuses.

In 2002, the ABS adopted an alternate method based on an approach by Bhat to estimate Aboriginal denominator populations and account for migration effects and increasing rates of Aboriginal self-identification at each census [25]. This method was a re-formulation of the General Growth Balance (GGB) model, first developed by Hill to account for migration [26]. The re-formulation aimed particularly to adjust for the larger problem of changing rates of Aboriginal identification at the census, referred to as “unexplained population growth,” which were treated as migration effects. The estimates of Aboriginal LE from the resulting “experimental Aboriginal life tables” were 17–19 years below the population overall [27]. An underlying problem with the Bhat method, however, was that intercensal Aboriginal population growth rates had to be estimated, which in part depended on knowing Aboriginal mortality in the first place, a circularity that undermines this approach [14,28].

Aboriginal life expectancies were estimated using the original GGB approach in the Burden of Disease and Injury in Aboriginal and Torres Strait Islander Peoples 2003 study [14]. Increasing rates of Aboriginal self-identification were treated as changes in census coverage, rather than migration effects, which do not assume a constant increase over time but nonetheless are assumed to be constant across age groups [14]. Using the GGB approach, the estimated Aboriginal LE for 1996–2001 was around 64 years for males and 69 for females, 11–12 years lower than for Australia overall [14,29]. Subsequent to this study, the ABS used a direct approach for its estimates of Aboriginal LE for 2005–2007 since recording Aboriginality at death registration had improved in the intervening period. The 2005–2007 ABS estimates were based on probabilistic linkage of the 2006 census records with death registrations between 9 August 2006 and 30 June 2007. Resulting LE estimates were 67 years for males and 73 years for females, 10–12 years below the overall Australian population [15].

The ABS estimate of the extent of Aboriginals identifying as such on death registration was approximately 85% after the linkage was performed [30]. However, under-coverage of Aboriginality in both population and death data remains [31,32]. The denominator relies on results of the census Post-Enumeration Survey (PES), which is compared with the census results. However, persons likely to avoid identification at the census are also likely to avoid identification in the PES. In approximately 26% of the records used in the ABS linkage project, Aboriginal status was not recorded. These records remained unlinked, thus potentially under-estimating Aboriginal mortality and over-estimating Aboriginal LE [31].

Another source of bias, which is a common problem with mortality studies of cohorts, and to a lesser extent with estimating mortality in whole populations, is that outmigration in the cohort or mortality occurring overseas has not been accounted for. For cohort studies, without a linkage with all death and population registers internationally (which is infeasible) or knowledge of movements abroad, bias in both the numerator and denominator from this source cannot be avoided. It is possible that some among the deaths known to the AMS but which failed to link with the NDS may have been of AMS clients who died abroad and whose death was not registered in Australia. However, the cohort is Australian-born and of Indigenous ethnicity who view Australia as their home country, and thus are less likely to migrate in middle or older age prior to death, as sometimes occurs in Australians born overseas. Finally, as the extent of out-migration of Aboriginal people is negligible [15], this source of bias is not expected to affect the estimates presented here substantially or significantly.

The main advantage of the population examined in the present study of AMS clients is that both the numerator and denominator are definitely Aboriginal people, so that uncertainties of designation of Aboriginality by third parties, as happens with death registration (numerator), or reluctance to nominate as Aboriginal or Torres Strait Islander in first person at the census (denominator), are absent from the mortality and LE estimates of the present study.

Life expectancy in the AMS Redfern cohort was similar to the GGB model and ABS estimates for Aboriginal people nationally, but somewhat lower than the GGB and ABS estimates for NSW Aboriginal people, significantly so in males. Also, in females the time trend of increasing LE in AMS cohort females was not as parallel with the more steeply rising trend in GGB and ABS estimates, as it was for the males. While it is difficult to compare secular trends in Aboriginal LE and mortality – due to changing rates of Aboriginal recording at death registration and in the census, and also to the different methodologies used to estimate these – the LE trends in the AMS males and females closely parallel those in the corresponding Australian male and female populations (Figure 4).

While the comparability of trends in LE may lend credence to the estimates presented here, and indicates that LE improvements in metropolitan Aboriginal people are apparent and have occurred in line with the general population, the absolute levels of life expectancy estimated from the AMS cohort are significantly lower. A number of biases limit the generalisability of the mortality estimates from the AMS cohort. The AMS client population is self-selected by longevity and by the high profile of the AMS in the Aboriginal community. The AMS client base includes disproportionately high proportions of prominent Aboriginal people in the arts, sciences, industry, commerce, academia, law, government, and politics. Such a population characteristic is likely to overstate the life expectancy of a geographically similar Aboriginal population. Moreover, it is highly probable that additional Aboriginal deaths have occurred in the cohort despite the extensive linkage work performed. This would also bias estimates of LE upwards. Without exact information on the extent of these, nor their age and sex distribution, it is difficult to perform a sensitivity analysis assuming different levels of under-enumeration of deaths.

The proportion of the AMS Redfern cohort aged 0–14 years was 19%, compared to 38% of Aboriginal people in NSW based on the 2006 Australian census [8]; those aged 25–44 years comprised 40% of the AMS cohort, compared to 25% in the census. These age distributions are rather different but their effect on age-standardised mortality and LE estimates would not be substantial. Direct age standardisation and LE calculations depend only on age-specific mortality rates to produce expected death numbers in a standard population (direct age standardisation) or in a hypothetical cohort (LE). Therefore, despite the difference in age distribution between the AMS Redfern cohort and the NSW Aboriginal population, the estimates of mortality and LE derived here are not substantially affected by bias from this source.

This is the first empirical study of mortality in a substantial sample of Aboriginal people in NSW. Mortality reductions in the AMS Redfern cohort have occurred mainly in younger age groups, and while life expectancy is estimated to be 11–12 years below the Australian and NSW populations, it appears to have increased over 1995–2009 in line with that of the Australian population overall.

## Competing interest

The authors declare that they have no competing interests.

## Authors’ contributions

SM performed the analyses and drafted the paper. RT, SM and JD conceived the study. BP supervised the data linkage and performed the clerical review of linked medical and death records to ascertain certainty of matches, and critically reviewed drafts of the manuscript. RT critically reviewed and edited drafts of the manuscript. JD critically reviewed drafts of the manuscript. KB critically reviewed drafts of the manuscript. NM critically reviewed drafts of the manuscript. All authors read and approved the final manuscript.
